# Analysis of Prevalence and Risk Factors of Adult Self-Reported Allergic Rhinitis and Asthma in Plain Lands and Hilly Areas of Shenmu City, China

**DOI:** 10.3389/fpubh.2021.749388

**Published:** 2022-01-04

**Authors:** Huiping Gao, Yongliang Niu, Qiang Wang, Guangliang Shan, Chao Ma, Haiying Wang, Yaoda Hu, Kai Guan, Jianqing Gu, Jing Wang, Tao Wang, Hongmei Zhao, Hui Han, Haiyuan Chen, Wenxia Ruan, Hanlin Zhang, Cong Cong, Lianglu Wang, Yonglin Liu

**Affiliations:** ^1^Shenmu Hospital, The Affiliated Shenmu Hospital of Northwest University, Shenmu, China; ^2^School of Basic Medicine, Institute of Basic Medical Sciences, Peking Union Medical College, Chinese Academy of Medical Sciences, Beijing, China; ^3^Department of Allergy, Peking Union Medical College Hospital, Chinese Academy of Medical Sciences, Beijing, China

**Keywords:** allergic rhinitis, asthma, prevalence, risk factors, plain lands and hilly areas

## Abstract

**Objective:** The main aim of this study was to investigate the prevalence and risk factors of adult self-reported allergic rhinitis and asthma in plain lands and hilly areas of Shenmu City in China, and analyze the differences between regions.

**Methods:** The multi-stage stratified random sampling was applied in a cross-sectional survey of adult residents in Shenmu City, from September to December 2019. The unconditional logistic regression analysis was used to screen the influence factors of allergic rhinitis and asthma.

**Results:** 4,706 adults participated in the survey, and 99% (4,655 in 4,706) completed the questionnaires. The prevalence of allergic rhinitis was 25.4%, and the prevalence of asthma was 9.4%. The prevalence of the allergic rhinitis without asthma, asthma without allergic rhinitis, and the combined allergic rhinitis with asthma were 18.9, 2.9, and 6.5%, respectively. The prevalence of allergic rhinitis and asthma existed regional differences. The prevalence of adult self-reported allergic rhinitis was 41.5% in plain lands areas and 22.1% in hilly areas. The prevalence of adult self-reported asthma was 12.8% in plain lands and 8.8% in hilly areas. The prevalence of allergic rhinitis and asthma existed seasonal differences, with the highest prevalence from July to September. The analysis of risk factors showed that higher education [middle and high school (OR 1.72, 95%CI 1.42–2.07); college and above (OR 2.67, 95%CI 1.99–3.59)], comorbidities of other allergic diseases (OR 3.90, 95%CI 3.23–4.70), family history of allergies (OR 2.89, 95%CI 2.36–3.53), and plain lands areas (OR 2.51, 95%CI 2.06–3.05) were the risk factors for the allergic rhinitis without asthma. Aging [40–49 years old (OR 4.29, 95%CI 1.02–18.13); 50–59 years old (OR 5.89, 95%CI 1.40–24.76); ≥60 years old: (OR 6.14, 95%CI 1.41–26.71)], never-smokers (OR 1.66, 95%CI 0.99–2.80), comorbidities of other allergic disorders (OR 2.17, 95%CI 1.42–3.32), and family history of allergies (OR 2.20, 95%CI 1.40–3.47) were the risk factors for the asthma without allergic rhinitis. Advanced age [30–39 years (OR 2.16, 95%CI 1.23–3.82); 40–49 years (OR 2.86, 95%CI 1.56 to 5.25); 50–59 years (OR 2.95, 95%CI 1.58–5.51); ≥60 years old (OR 2.27, 95%CI 1.09–4.72)], higher education [middle and high school (OR 2.23, 95%CI 1.62–3.07); college and above (OR 4.28, 95%CI 2.72–6.74)], non-agricultural workers (OR 1.70, 95%CI 1.18–2.43),never-smokers (OR 2.26, 95%CI 1.51–3.39), comorbidities of other allergic diseases (OR 4.45, 95%CI 3.37–5.88), family history of allergies (OR 5.27, 95%CI 3.98–6.97), and plain lands areas (OR 2.07, 95%CI 1.51–2.86) were the risk factors for the combined allergic rhinitis with asthma.

**Conclusions:** The prevalence of allergic rhinitis and asthma in Shenmu City was relatively high, with regional differences. Genetic and environmental factors were the important risk factors associated with allergic rhinitis and asthma. Our research would provide data support for preventing and controlling allergic rhinitis and asthma in this region in the future, and appropriate prevention and control programs should be formulated according to the characteristics of different regions.

## Introduction

Allergic rhinitis (AR) and asthma, as one of the common chronic diseases in the world, have become a health problem that cannot be ignored. It is estimated that nearly 500 million and 300 million (1) people worldwide suffer from allergic rhinitis and asthma respectively, and the incidence is on the rise. A number of surveys have shown that the prevalence of the two diseases varies in different countries and regions. China is the most populous country in the world. In recent years, with the gradual development of epidemiological investigation and in-depth research, it has been found that the prevalence of AR and asthma in China has been increasing significantly. The results of two surveys conducted by Zhang and his team in Beijing Tongren Hospital in 2005 and 2011 respectively showed that the self-reported prevalence of AR increased from 11.1 to 17.6% in 6 years (2). The findings of Lin et al. from 2010 to 2012 showed that the prevalence rate of asthma in patients >20 years old in mainland China was 1.29% (3). In 2015, Wang et al. found that the prevalence rate of asthma in Chinese adults aged 20–80 was 4.2% (4). Smoking, indoor and outdoor air pollution, occupational exposures, allergens and overweight are major risk factors associated with asthma, while house allergens, smoke, pollen, food, mites, odors and fumes are the risk factors associated with allergic rhinitis (5, 6).

China has a vast territory, and there are great differences in geographical features, climate conditions, economic conditions and living habits among different regions, leading to obvious regional differences in the prevalence of AR and asthma in different regions of China. Current cross-sectional studies based on a number of natural populations show that the prevalence of AR ranges from 8.7% in Beijing to 32.4% in the grassland areas of northern China (7, 8). The prevalence of asthma also ranged from 0.5 to 4.2% (4, 9). Studies have shown that AR prevalence presents regional characteristics. In Inner Mongolia Autonomous Region, the AR prevalence rate in Baotou city was 7.96% (10), the confirmed AR prevalence rate in Tongliao city was 18.1% (11), and the AR prevalence rate in Xilinhot city was 52.9% (8), suggesting that it is related to the landform, vegetation distribution, and pollen species and concentration in this region to a certain extent. Shenmu city, located in the north of Yulin City, is the junction of Mongolia, Shaanxi and Ningxia provinces, and is located in the transition zone from Mu us Plain lands to loess hilly area of northern Shaanxi province. It belongs to the interlacing area of agriculture and animal husbandry, and its ecological environment is fragile and sensitive to change. In recent years, under the background of “sand retreat and afforestation,” vegetation grade has changed. The number of AR cases has increased significantly, especially seasonal allergic rhinitis, which has greatly affected the lives and work of local residents. However, there is a lack of investigation on the prevalence of AR in this region.

With the strong support of Shenmu City Government funds, we established a scientific research team in 2018. Led by Shenmu City Hospital, and with the cooperation of The Institute of Basic Medical Sciences of Chinese Academy of Medical Sciences and the Allergy Department of Peking Union Medical College Hospital, we completed the first cross-sectional study based on large samples of natural population in Shenmu city. This study intends to carry out an epidemiological survey of self-reported AR in Shenmu City, in order to understand the prevalence of AR in the local population, and to understand the main risk factors of AR and asthma in different areas combined with the characteristics of sandstorm grass beach and loess hill, so as to provide reliable epidemiological data for the prevention and controlling of allergic diseases in this region and the subsequent cohort study.

## Methods

### Research Area and Research Object

**Figure 1:**
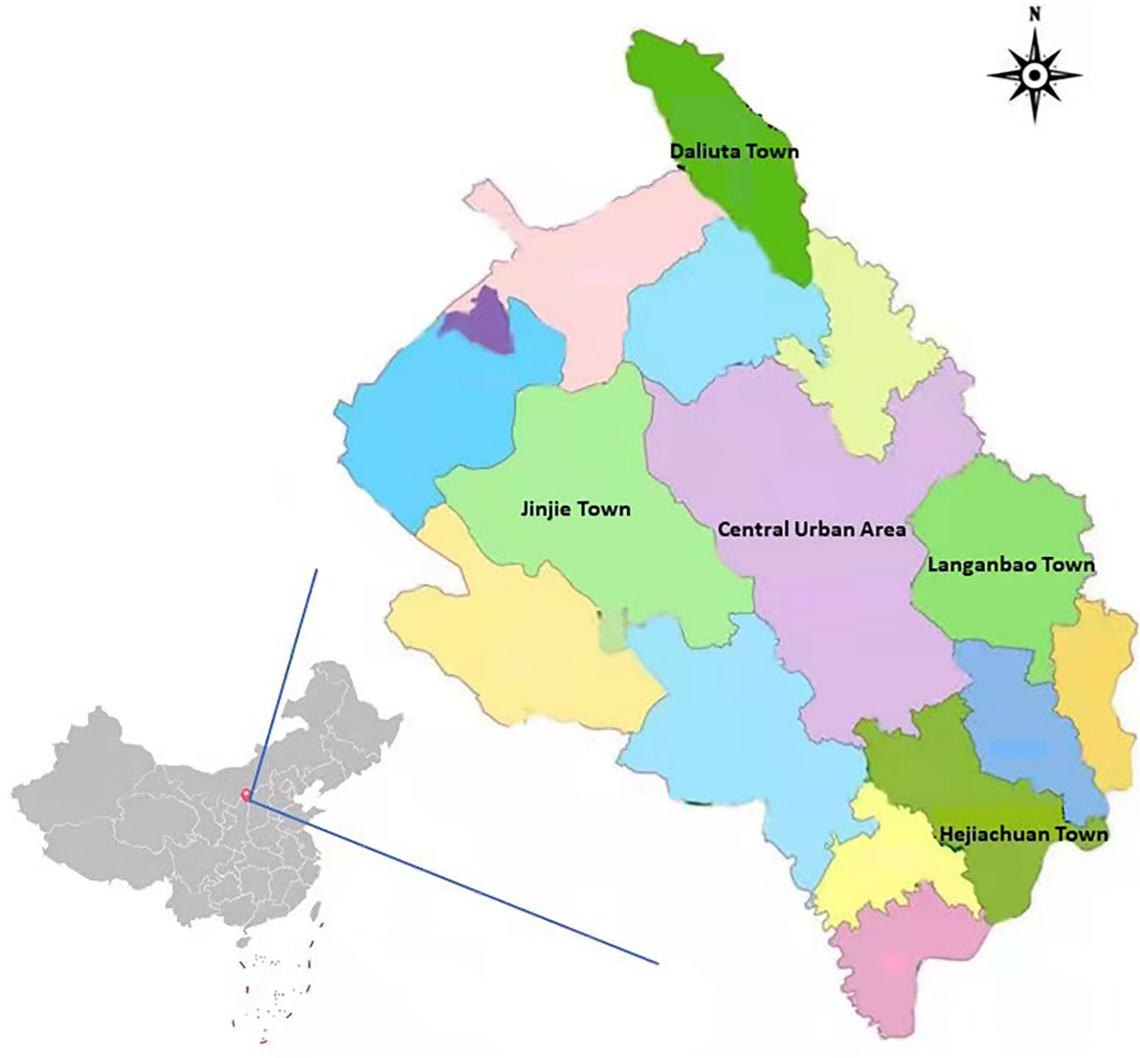
Location of five survey sites in Shenmu City.

This study was conducted in Shenmu City along the Great Wall, the middle reaches of the Yellow River and southeast of the Mu us Plain lands in Shaanxi Province. The city is located between 38°13′-39°27′ north latitude and 109°40′-110°54′ east longitude, high in the northwest and low in the southeast, the altitude of the city ranges from 738.7 to 1,448.7 m. With a total area of 7635 km^2^, the city is the largest county in Shaanxi Province. It has jurisdiction over 14 towns, 6 sub-district offices and 326 administrative villages with a total population of 548,000. The cross-sectional study was conducted from August 2019 to December 2019. This survey adopted multi-stage stratified random sampling method to select 5 towns in Shenmu City as survey points, including Daliuta Town in plain lands area, Jinjie Town, Langanbao Town, Hejiachuan Town and central urban area in hilly areas, and we randomly select Linzhou street and Yingbin Road street as representatives of central urban area. Detailed survey sites and geomorphic distribution were shown in Figure 1.

The study investigated adult residents (aged 18 and above) with Han ethnicity permanent residents (defined as those who have lived in the survey area for at least one year) in Shenmu City on the household basis. The calculation of sample size was based on an estimated prevalence of asthma that is 8 % in Shenmu city. To reach the significance level of 0.05 and error tolerance of 0.1 × p, the estimated minimum sample size was 4,600, and further considering the 5% non-response rate 4,842 cases need to be investigated. According to the prevalence rate of allergic rhinitis 11.42% (12), 3,267 cases need to be investigated. This study actually investigated 4,706 people, excluding those who did not receive the questionnaire for allergic rhinitis and asthma, and finally included 4,655 subjects for analysis, which met the minimum sample size. Jinjie Town and Daliuta Town were investigated in Jinjie Town Health Center and Daliuta Town Health Center respectively, and the other four investigation sites were conducted in Shenmu City hospital.

### Interviewer-Administered Questionnaire

The questionnaire format referred to the International Study on Asthma and Allergy in Childhood (ISAAC) and the European Community Respiratory Health Survey (ECRHS) (13, 14). Based on ISAAC and ECRHS, we revised and designed the online questionnaire format. The content of the online questionnaire included demographic factors (gender, age, education, occupation), smoking, the comorbidities of other allergic disorders (food allergy, drug allergy, skin allergy, urticaria, etc.), family history of allergies (allergic rhinitis, asthma, or eczema), and the place of residence. Besides, the online questionnaire contained preliminary screening for allergic rhinitis and asthma. All participants signed the written informed consent.

### Definitions of Self-Reported Allergic Rhinitis and Asthma

Definition of allergic rhinitis: according to the Allergic Rhinitis and Its Impact on Asthma (ARIA), the subjects with at least two of the four symptoms (itchy nose, sneezing, runny and blocked nose) in the previous 12 months after exposure to pollen, house dust mites or other allergens were considered as self-reported allergic rhinitis (15).

Definition of asthma: Based on the ECRHS questionnaire, the subjects with at least one of the following questions choosing ” Yes “ were considered as self-reported asthma (13). Question 1. Have you ever experienced a whistling or hissing sound in your chest when you breathe or exhale? Question 2. In the past 12 months, have you experienced whistling wheezing or hissing in your chest when you breathe or exhale? Question 3. Do you feel chest tightness or shortness of breath during rest? Question 4. Have you ever been awake at night due to chest tightness and shortness of breath? Question 5. Have you experienced whistling wheezing or hissing in chest after doing exercises or physical activities? Question 6. Have you experienced the dry cough at night rather than getting cold? Question 7. Have you ever diagnosed as asthma?

Definition of the variables used to assess the seasonal variability: Based on the ECRHS questionnaire, the trend of allergic rhinitis and asthma symptoms changing with time was analyzed. Question 1. In the past 12 months, which months did these nasal symptoms occur? Question 2. Are the symptoms of wheezing and suffocation you suffered related to the season? If yes, please indicate the time of onset?

Considering the severity of the disease and the different prevention and treatment management strategies, this study further divided the patients with allergic rhinitis and asthma into four groups: disease-free group, the allergic rhinitis without asthma, the asthma without allergic rhinitis, and the combined allergic rhinitis with asthma. The disease-free group was taken as the reference group.

### Definition of Independent Variable and Grouping

Smoking status was divided into smoking and never smoking. Smoking included current and previous smoking. Current smoking was defined as smoking at least one cigarette per day for more than half a year, and previous smoking was defined as stopping smoking for at least 1 month during the survey. Never smoking was defined as having never smoked in the past. Other allergic diseases included food allergies, drug allergies, skin allergies, urticaria, etc. Family history referred to family history of allergic rhinitis, asthma, or eczema.

### Data Collection and Quality Control

In this study, a unified investigation process manual was developed to explain the specific process and notes of the entire investigation. Before the on-site investigation, the staff designated by local government introduced the project background and informed the investigation time and place to the subjects through telephone or wechat. All participants in the investigation were required to carry their ID cards and input their identity information on the spot. After centralized training and assessment, only qualified investigators can carry out on-site face-to-face electronic questionnaire survey. In order to reduce the survey bias, the investigators were fixed and all were professional medical staffs from Shenmu City Hospital. The electronic questionnaires are stored and backed up in the cloud, and the quality of questionnaires were reviewed by special personnel. Incomplete questionnaires were regarded as invalid and removed. The data with large deviation of information was further verified and confirmed by telephone, and the wrong data was corrected.

### Statistical Analysis

Medical dimension cloud scientific research big data platform is used for data entry. Based on the data type and distribution, the continuous variables which obey the normal distribution were described by the mean ± standard deviation, and the categorical variables were described by the frequency (percentage). T-test and Chi-square test were used to compare the differences between groups. In this study, unconditional logistic regression was used for univariate analysis of allergic rhinitis alone, asthma alone and allergic rhinitis combined with asthma. The disease-free group was taken as the reference group. Then the stepwise regression method was used to carry out multi-factor logistic regression analysis, with the set criterion for entry into model (SLE) 0.20 and the set criterion for staying in the model (SLS) 0.05. The odds ratio (OR) and 95% confidence interval (95% CI) of related factors were also obtained. *P* < 0.05 was considered statistically significant. All statistical analysis was completed by SPSS 26.0.

## Results

### Demographic Characteristics of Survey Subjects

The average age of the 4,655 respondents in this survey was 47.30 ± 11.34 years old. 1,866 (40.1%) were males, and 2,789 (59.9%) were females. After stratified by different landforms, the proportion of males in the hilly area was higher than that in the plain lands areas (*P* < 0.001) (Table 1). In addition, the age of the population surveyed in the hilly area was slightly higher than that in the plain lands area (47.45 ± 11.25 vs. 46.56 ± 11.80, *P* = 0.044). Compared with hilly areas, the demographic characteristics of people living in plain lands areas presented higher education level (*P* < 0.05), lower smoking rate (*P* < 0.05), and higher family history rate of other allergic disorders (*P* < 0.05) (Table 1). However, there was no significant difference in the amount of smoking and the number of years of smoking between people living in plain lands areas and those living in the hilly areas (Table 1).

**Table 1:** Demographic characteristics of the participants.

**Variables**	**Total**	**Plain lands areas**	**Hilly areas**	***P* value**
**Total**	4,655	784	3,871	
**Men**, ***n*** **(%)**	1,866 (40.1)	261 (33.3)	1,605 (41.5)	<0.001
**Age (years)**, ***n*** **(%)**				0.095
18–29	320 (6.9)	70 (8.9)	250 (6.5)	
30–39	927 (19.9)	159 (20.3)	768 (19.8)	
40–49	1,314 (28.2)	211 (26.9)	1,103 (28.5)	
50–59	1,345 (28.9)	230 (29.3)	1,115 (28.8)	
≥60	749 (16.1)	114 (14.5)	635 (16.4)	
**Educational level**, ***n*** **(%)**				<0.001
Primary school and lower	2,338 (50.2)	384 (49.0)	1,954 (50.5)	
Middle and high school	1,719 (36.9)	241 (30.7)	1,478 (38.2)	
College and higher	598 (12.8)	159 (20.3)	439 (11.3)	
**Cigarette smoking**, ***n*** **(%)**				<0.001
Smoker	1,708 (36.7)	235 (30.0)	1,473 (38.1)	
Never-smoker	2,947 (63.3)	549 (70.0)	2,398 (61.9)	
Smoking years*, ***n*** **(%)**				0.482
≤ 20	569 (33.3)	83 (35.3)	486 (33.0)	
> 20	1,139 (66.7)	152 (64.7)	987 (67.0)	
Cigarettes per day*, ***n*** **(%)**				
≤ 20	1,463 (85.7)	208 (88.5)	1,255 (85.2)	0.179
> 20	245 (14.3)	27 (11.5)	218 (14.8)	
**Comorbidities of other allergic disorders**, ***n*** **(%)**				<0.001
Yes	846 (18.2)	184 (23.5)	662 (17.1)	
No	3,809 (81.8)	600 (76.5)	3,209 (82.9)	
**Family history of allergies**, ***n*** **(%)**				0.002
Yes	745 (16.0)	154 (19.6)	591 (15.3)	
No	3,910 (84.0)	630 (80.4)	3,280 (84.7)	
**Occupation**, ***n*** **(%)**				<0.001
Agricultural	1,316 (28.3)	269 (34.3)	1,047 (27.0)	
Non-agricultural	3,339 (71.7)	515 (65.7)	2,824 (73.0)	

*Compare the differences between the two groups only among smokers.

### Prevalence of Allergic Rhinitis and Asthma

**Figure 2:**
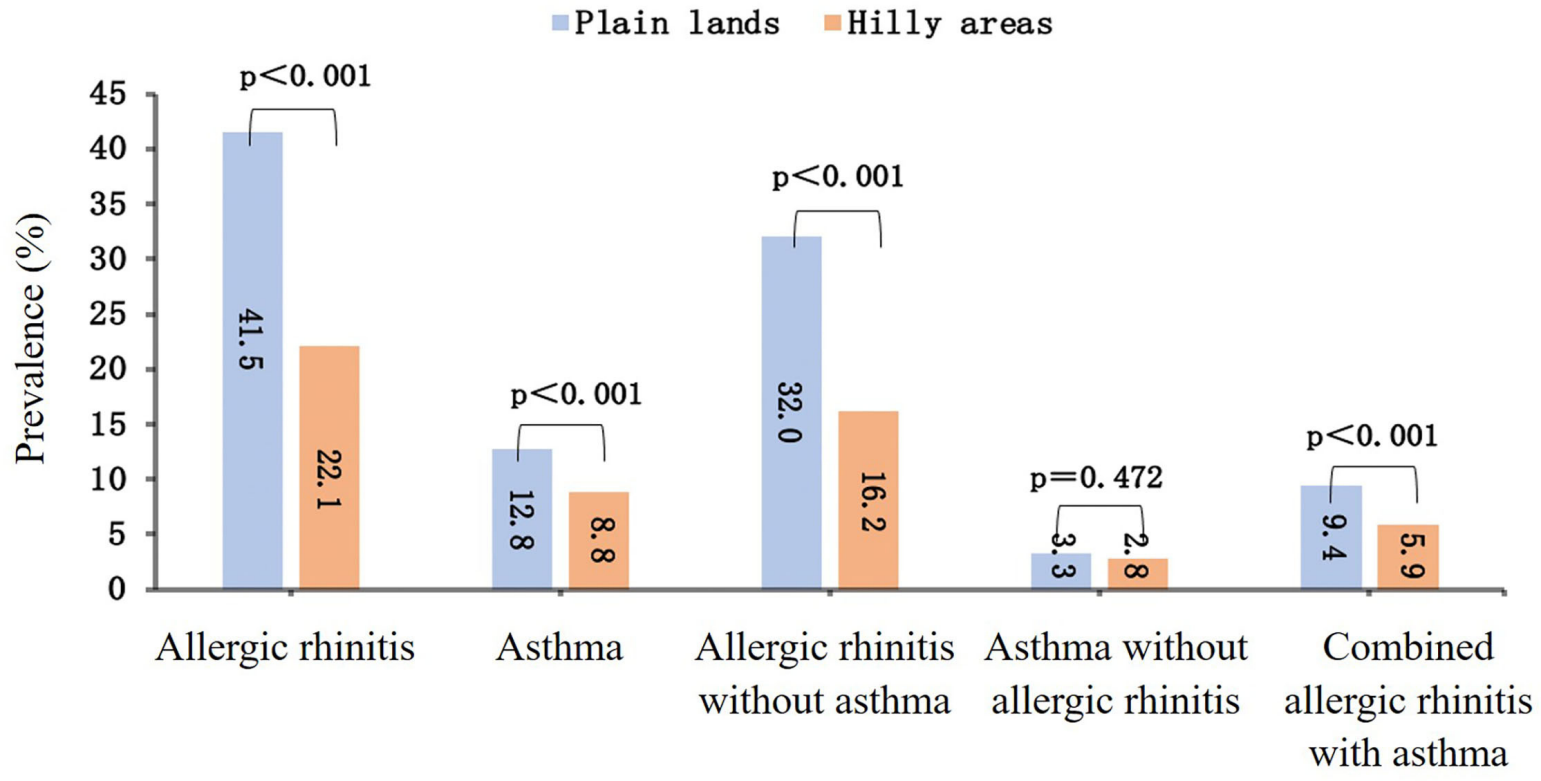
The prevalence of allergic rhinitis, asthma, allergic rhinitis without asthma, asthma without allergic rhinitis, and combined allergic rhinitis with asthma in plain lands and hilly areas.

The adult self-reported prevalence of allergic rhinitis was 25.4% (1,182/4,655), and the prevalence of asthma was 9.4% (439/4,655). Stratified by landscape, the prevalence of both allergic rhinitis and asthma was higher in the plain lands areas than that in the hilly areas: the prevalence of allergic rhinitis in the two regions was 41.5 and 22.1% (*P* < 0.001), and asthma was 12.8 and 8.8% (*P* < 0.001). Further divided by disease type, there were 879 people (18.9%) suffering from the allergic rhinitis without asthma, 136 people (2.9%) suffering from the asthma without allergic rhinitis, and 303 people (6.5%) suffering from the combined allergic rhinitis with asthma. According to the landscape stratification, the prevalence rate was calculated based on the total population in the plain lands and hilly areas. Compared with hilly areas, the prevalence of the allergic rhinitis without asthma, the asthma without allergic rhinitis, and the combined allergic rhinitis with asthma were significantly higher in plain lands areas (*P* < 0.05). The prevalence of different outcomes in different landforms was shown in Figure 2.

**Figure 3:**
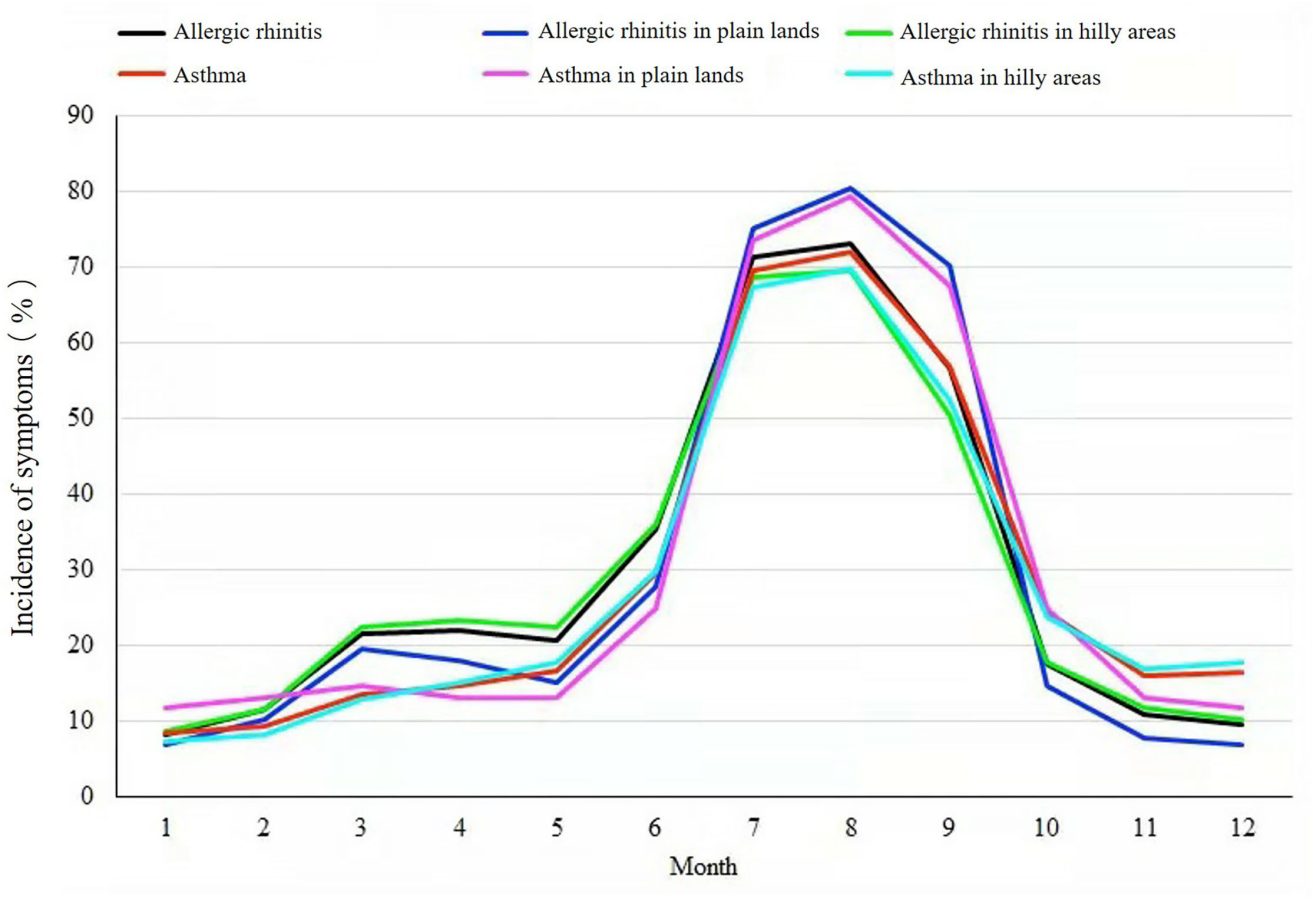
Landform variation, seasonal variation, and incidence of symptoms of allergic rhinitis and asthma.

In addition, the allergic rhinitis and asthma symptoms also changed over time, as shown in Figure 3. The highest prevalence of allergic rhinitis and asthma appeared from July to September. It was also observed that the symptoms of allergic rhinitis are more frequent from January to June, while the symptoms of asthma are more frequent from October to December. This phenomenon exists in both plain lands and hilly areas. The symptoms of allergic rhinitis and asthma were higher in the plain lands from July to September, while higher in the hilly area in other months.

### Risk Factors Analysis

This study analyzed the effects of totally eight factors including demographics (gender, age section, cultural degree, professional category), smoking status, history of allergic disease and family history (other allergic diseases, family history), living environment (landform category), on the allergic rhinitis without asthma, the asthma without allergic rhinitis, and the combined allergic rhinitis with asthma. Taking the disease-free group as reference, the single-factor analysis results (Table 2) showed that the prevalence of people that live in plain lands areas suffering from the allergic rhinitis without asthma was 2.06 times (95% CI: 1.83–2.33) higher than that prevalence of people living in hilly areas. Besides, the prevalence of the allergic rhinitis without asthma decreased along with aging (*P* < 0.001), whereas that prevalence increased along with a higher education level (*P* < 0.001). Moreover, never-smokers, non-agricultural workers, those with the comorbidities of other allergic disorders and with the family history of allergies were more likely to have allergic rhinitis without asthma.

**Table 2:** Risk factor analysis of the allergic rhinitis without asthma, asthma without allergic rhinitis, and combined allergic rhinitis with asthma.

**Variables**	**Allergic rhinitis**	***P* value**	**Asthma without**	***P* value**	**Combined allergic**	***P* value**
	**without asthma**		**allergic rhinitis**		**rhinitis with asthma**	
**Gender**, ***n*** **(%)**		0.879		0.013		0.021
men	361 (21.0)		41 (2.9)		103 (7.0)	
women	518 (20.8)		95 (4.6)		200 (9.2)	
**Age (years)**, ***n*** **(%)**		<0.001		0.008		<0.001
18–29	108 (36.6)		2 (1.1)		23 (11.0)	
30–39	223 (26.8)		14 (2.2)		81 (11.7)	
40–49	234 (19.8)		37 (3.8)		94 (9.0)	
50–59	205 (16.9)		54 (5.1)		81 (7.5)	
≥60	109 (15.7)		29 (4.7)		24 (3.9)	
**Educational level**, ***n*** **(%)**		<0.001		0.546		<0.001
Primary school and lower	316 (14.6)		80 (4.2)		96 (4.9)	
Middle and high school	355 (23.0)		47 (3.8)		129 (9.8)	
College and higher	208 (40.7)		9 (2.9)		78 (20.5)	
**Occupation**, ***n*** **(%)**		<0.001		0.057		<0.001
Agricultural	196 (16.1)		52 (4.9)		49 (4.6)	
Non-agricultural	683 (22.8)		84 (3.5)		254 (9.9)	
**Cigarette smoking**, ***n*** **(%)**		<0.001		0.007		<0.001
Smoker	286 (17.9)		38 (2.8)		70 (5.1)	
Never-smoker	593 (22.7)		98 (4.6)		233 (10.3)	
**Comorbidities of other allergic disorders**, ***n*** **(%)**		<0.001		<0.001		<0.001
Yes	319 (46.4)		31 (7.8)		128 (25.8)	
No	560 (15.9)		105 (3.4)		175 (5.6)	
**Family history of allergies**, ***n*** **(%)**		<0.001		0.001		<0.001
Yes	256 (43.6)		26 (7.3)		132 (28.5)	
No	623 (17.2)		110 (3.5)		171 (5.4)	
**Landform**, ***n*** **(%)**		<0.001		0.038		<0.001
Plain lands areas	251 (36.7)		26 (5.7)		74 (14.6)	
Hilly areas	628 (17.8)		110 (3.6)		229 (7.3)	

The prevalence of people that live in plain lands areas suffering from asthma without allergic rhinitis was 1.55 times (95% CI: 1.02–2.35) higher than that prevalence of people living in hilly areas. Different from the allergic rhinitis without asthma, the prevalence of the asthma without allergic rhinitis increased along with aging (*P* <0.05), whereas that prevalence decreased along with a higher education level. For the combined allergic rhinitis with asthma, the prevalence of people living in plain lands areas was 2.00 times (95% CI: 1.56–2.55) higher than that in hilly areas. Except for the fact that women are more likely to develop allergic rhinitis with asthma, other features are similar with the allergic rhinitis without asthma.

Table 3 showed the results of multivariate analysis. Overall, risk factors including education, the comorbidities of other allergic diseases, family history of allergies, and the place of residence would affect the prevalence of the allergic rhinitis without asthma. After adjusting for other factors, compared with people in the hilly area, the risk of suffering from the allergic rhinitis without asthma increased 251% (95% CI: 206–305%) in people in the plain lands areas. Besides, aging, smoking, the comorbidities of other allergic diseases, and family history of allergies were the risk factors that affected the prevalence of asthma without allergic rhinitis, whereas the place of residence had no statistical significance for its prevalence after adjusting for other factors. In the process of multivariate analysis, aging, education, occupation, smoking, the comorbidities of other allergic diseases, family history of allergies, and the place of residence were the risk factors for the combined allergic rhinitis with asthma. After adjusting for other factors, the prevalence of people who live in plain lands areas suffering from the combined allergic rhinitis with asthma was 2.07 times (95% CI: 1.51–2.86) higher than that in hilly areas.

**Table 3:** Multivariate logistic regression analysis of risk factors for the allergic rhinitis without asthma, asthma without allergic rhinitis, and combined allergic rhinitis with asthma.

**Variables**	**Allergic rhinitis without asthma**				**Asthma without allergic rhinitis**				**Combined allergic rhinitis with asthma**			
	**β**	**Standardized β**	**OR (95%CI)**	***P***	**β**	**Standardized β**	**OR (95%CI)**	***P***	**β**	**Standardized β**	**OR (95%CI)**	***P***
**Age (years)**												
18–29			NA				1				1	
30–39	NA	NA	NA	NA	0.765	0.874	2.40 (0.54–10.70)	0.252	0.087	0.772	2.16 (1.23–3.82)	0.008
40–49	NA	NA	NA	NA	1.293	1.457	4.29 (1.02–18.13)	0.047	−0.216	1.052	2.86 (1.56–5.25)	0.001
50–59	NA	NA	NA	NA	1.614	1.774	5.89 (1.40–24.76)	0.015	−0.423	1.082	2.95 (1.58–5.51)	0.001
≥60	NA	NA	NA	NA	1.530	1.815	6.14 (1.41–26.71)	0.015	−1.101	0.820	2.27 (1.09–4.72)	0.028
**Educational level**												
Primary school and lower			1				NA				1	
Middle and high school	0.557	0.540	1.72 (1.42–2.07)	<0.001	NA	NA	NA	NA	0.736	0.803	2.23 (1.62–3.07)	<0.001
College and higher	1.389	0.984	2.67 (1.99–3.59)	<0.001	NA	NA	NA	NA	1.599	1.454	4.28 (2.72–6.74)	<0.001
**Occupation**												
Agricultural			NA				NA				1	
Non-agricultural	NA	NA	NA	NA	NA	NA	NA	NA	0.824	0.528	1.70 (1.18–2.43)	0.004
**Cigarette smoking**												
Smoker			NA				1				1	
Never-smoker	NA	NA	NA	NA	0.516	0.509	1.66 (0.99–2.80)	0.056	0.771	0.814	2.26 (1.51–3.39)	<0.001
**Comorbidities of other allergic disorders**												
No			1				1				1	
Yes	1.525	1.360	3.90 (3.23–4.70)	<0.001	0.868	0.776	2.17 (1.42–3.32)	<0.001	1.775	1.492	4.45 (3.37–5.88)	<0.001
**Family history of allergies**												
No			1				1				1	
Yes	1.317	1.060	2.89 (2.36–3.53)	<0.001	0.764	0.789	2.20 (1.40–3.47)	0.001	1.947	1.662	5.27 (3.98–6.97)	<0.001
**Landform**												
Hilly areas			1				NA				1	
Plain lands areas	0.986	0.918	2.51 (2.06–3.05)	<0.001	NA	NA	NA	NA	0.773	0.729	2.07 (1.51–2.86)	<0.001

*In a multifactor model based on SLE = 0.2 and SLS = 0.05, NA indicates that the factor does not meet the above criteria and is not included in the final model*.

## Discussion

This study is the first regular population based epidemiological survey about allergic rhinitis and asthma in Shenmu City. The results of the survey showed that the prevalence of self-reported allergic rhinitis and asthma among adults in Shenmu City was higher than previously reported studies. There were significant seasonal and topographical differences, in symptoms and prevalence of AR and asthma. In addition, this study also analyzed the risk factors related to allergic diseases, aiming to provide reference for the prevention and control of allergic diseases in the region and different geographies in northern China in the future.

The prevalence of AR in this study is lower than that 30.2–41.3% of Western developed countries (16–20), which may be related to the level of economic development. The results of Brozek et al. showed that the prevalence of AR varies with the economy and the prevalence rate showed an obvious upward trend related to economic advancement (21). Meanwhile, residents in western developed countries are more likely to obtain medical care services and the detection rate may be higher (22, 23). Although the results in this study are consistent with the previous AR survey results in China (6.24–32.4%), there are still obvious regional differences. For example, the prevalence of AR in this study is higher than that in East China (12.08% in Qingdao, 16.47% in Ningbo), South China (6.24% in Guangzhou), and Northeast China (14.12% in Liaoning Province), but lower than that in North China (32.4% in Inner Mongolia) and northwestern China (30.04% of Xinjiang) (24). These regional differences might be due to different geographical distribution of vegetation and pollen. A survey conducted in 2015 (12) indicated that the prevalence of AR has been on the rise in recent years, which may be related to the ecological work of “desert greening” i.e., forest plantation in large sand areas. For this purpose, a large number of vegetations such as Artemisia sphaerocephala, Talang, Hadawang, and Huabang florae have been planted during 1958–2015. These vegetations produce a large amount of Artemisia pollen, which may increase the sensitivity of individuals exposed to this type of pollen. Besides, our study also showed that the prevalence of asthma is significantly higher than the national average, probably because only lung function test was considered in the national survey, while our study was based on the diagnosis of asthma symptoms. This might cause overestimated prevalence, but can identify high-risk groups that require further diagnosis and can also reduce chances of missed diagnosis, which has practical significance for disease prevention.

Our results show that whether it is AR or asthma, the prevalence in plain lands areas is higher than that in hilly areas. This might be because plain lands have more pollen, dust and house dust mites as compared to hilly areas (25, 26). Moreover, pollinator visitation rates to pollen flowers are also significantly higher in plain areas (27). Daliuta Township, as the representative of plain lands areas, is the industrial and mining economic zone of Shenmu City, where Artemisia, being widely planted to fix sand, is the unique local vegetation. The Artemisia pollen was confirmed as one of the most important allergenic pollens in summer and autumn in northern China (28). Results of pollen test revealed that the most abundant airborne pollen is Artemisia, accounting for as much as 78.24%, in Shenmu city (Ma et al., 2009). The Artemisia pollen was the absolute dominant airborne pollen in the region, with the pollen season from July to September (29). In our investigation, the prevalence of allergic rhinitis and asthma showed seasonal differences, with the highest prevalence from July to September. Previous studies have also reported seasonal variations of prevalence in asthma and allergic rhinitis cases respectively (30–32). We found that in the serum allergen sIgE test (including dust mite, Alternaria alternata, Artemisia and inhalation screening) the positive rate was 70.69%. The positive rate of Artemisia allergy in the self-reported AR ranged from 8.65% in the hilly area to 14.31% in plain lands area, which was consistent with the questionnaire survey results. Furthermore, we also noticed that compared with hilly areas, residents of plain lands had higher education level (*P* < 0.05). Reduced mobility and lesser transport opportunities might be the cause of lower rate of education in hilly areas. A study conducted in India also showed lesser education levels in hilly areas because of reduced mobility (33). Besides, lower smoking rate was also found in plain lands as compared to hilly areas (*P* < 0.05) which is in consistence with the study done in Nepal (34). These differences between the plain lands and hilly areas might also contribute to the different prevalence of AR or asthma. Based on these, it is necessary to carry out preventive interventions targeting high-risk populations in plain lands areas from July to September every year, including the popularization of preventive knowledge of allergic rhinitis and asthma and enhancement of the related medical system.

Our investigation identified four risk factors for the allergic rhinitis without asthma, four risk factors for the asthma without allergic rhinitis, and seven risk factors for the combined allergic rhinitis with asthma in Shenmu City. Among them, both the comorbidities of other allergic disorders and family history of allergies were the risk factors for the allergic rhinitis without asthma, asthma without allergic rhinitis, and the combined allergic rhinitis with asthma, respectively. Moreover, we found that the genetic susceptibility of the allergic rhinitis without asthma, the asthma without allergic rhinitis, and the combined allergic rhinitis with asthma were consistent with previous studies (35–37). It was reported that genetics plays an important role in predisposing eczema to AR, and particulate matter 2.5 (PM_2.5_) exposure can increase DNA methylation of IFN-γ gene promoter in CD4 + T cells. Single nucleotide polymorphisms (SNPs) as an important genetic factor can increase or decrease the susceptibility to AR via interleukin, chemokine, and receptor coding genes and play an important role in predisposing AR. FHL2 has also been discovered as a novel gene associated with heritability of AR. Epigenetic alterations including DNA methylation, histone modifications, and microRNAs (miRNAs) are involved in AR manifestation. Some miRNAs have been recognized as important regulators of allergic inflammation such as miRNA-155, miR-92b, miR-210, and miR-34a (38). Therefore, family inheritance probably existed in Allergic rhinitis and asthma. Furthermore, higher education and plain lands areas were risk factors for the allergic rhinitis without asthma and the combined allergic rhinitis with asthma, which might be related to the industrialization, landforms, and vegetation distribution of Shenmu City. In addition, the prevalence of the combined allergic rhinitis with asthma was highest between the age of 18 to 39, while the prevalence of asthma without allergic rhinitis increased with age, which was consistent with another asthma epidemiological study in Jinan City, China (39).

In our study, non-smokers were more likely to suffer from the allergic rhinitis without asthma, the asthma without allergic rhinitis, and the combined allergic rhinitis with asthma. Nevertheless, smoking was regarded as the risk factor for allergic rhinitis and asthma. Previous studies showed that smoking induced sensitization to many allergens, and smokers had higher levels of IgE, IgG4, and histamine in nasal lavage fluid (40–42). Also, compared with non-smoker asthma patients, smokers with asthma had worse lung function tests and more severe symptoms, and their asthma symptoms improved significantly after quitting smoking one year later (43). There are several possible explanations for this inconsistency. On one side, the proportion of women in non-smokers is significantly higher than that in smokers (85.6 and 15.5%). It is worth noting that the prevalence of allergic rhinitis in women in this study is higher than that in men (25.7 vs. 24.9%), which is consistent with the published results, that is elevated estrogen positively correlated with AR (44). On the other hand, patients with allergic rhinitis and asthma might start to quit smoking at the beginning of the onset (45). Because in our investigation, the smoking cessation rate of people with allergic rhinitis and asthma was 28.68%, while the smoking cessation rate of the healthy control was 16.74%, and the difference between the two was statistically significant (*P* < 0.001). Additionally, the low smoking rate was also the potential reason, considering 63.3% of the participants in this study had never smoked before. However, the specific reasons need further verification.

It has been proved the average age of the onset of allergic rhinitis caused by autumn pollen was significantly smaller than that of allergic asthma, and nearly half of patients with allergic rhinitis caused by autumn pollen developed seasonal allergic asthma within nine years (46–48). In fact, allergic rhinitis was an independent risk factor for the onset of asthma, and 40% of allergic rhinitis patients had asthma (49). The inflammatory responses of the upper and lower airways might be similar and affected each other. The prevalence of the combined allergic rhinitis with asthma was relatively high in this study. Future research should focus on the relationship and pathological process of allergic rhinitis and asthma, as well as developing methods to screen out the high-risk populations of allergic comorbidities, which would be helpful in disease prevention and control.

There are several limitations in our study. First of all, the epidemiological survey did not include residents under the age of 18, so it could not provide the whole picture regarding the prevalence in Shenmu City. Second, the diagnosis of allergic rhinitis and asthma was self-reported and based on the interviewer-administered questionnaire, without skin prick tests. Third, other risk factors such as different pollen types and air pollutants were not analyzed in our research. Finally, this study displayed that the symptoms of allergic rhinitis and asthma in the population surveyed are seasonal, but the seasonal rhinitis was not further distinguished, which should be analyzed in future.

In summary, our investigation was the first epidemiological study of allergic rhinitis and asthma in the plain lands and hilly areas of Shenmu City. We found the prevalence of self-reported allergic rhinitis and asthma were high in the plain lands and hilly areas, and the prevalence varied greatly due to industrialization, geographical location, the landform, and vegetation distribution. Besides, the prevalence of allergic rhinitis and asthma existed seasonal differences, with the highest prevalence from July to September. We also identified multiple risk factors for allergic rhinitis and asthma in this area. This study provides scientific basis for study of the prevention and control of allergic diseases in this region, and underlines an urgent need to develop routine screening and scientific intervention programs targeting high-risk groups, thereby guides local residents to improve the quality of life in the future.

## Data Availability Statement

The raw data supporting the conclusions of this article will be made available by the authors, without undue reservation.

## Ethics Statement

The studies involving human participants were reviewed and approved by Ethics Committee of Shenmu City Hospital (No.: sm004). The patients/participants provided their written informed consent to participate in this study. Written informed consent was obtained from the individual(s) for the publication of any potentially identifiable images or data included in this article.

## Author Contributions

HG, YL, and LW: conceptualization. HC, HG, and HW: data curation. HG and YL: formal analysis and writing-original draft. YL: funding acquisition. YN, HW, HH, and HC: investigation. HG, GS, YH, and YL: methodology. QW, YL, YN, LW, and CM: project administration. YL and LW: supervision. HH, HC, and WR: validation. GS, CM, YH, KG, JG, JW, HZhan, TW, CC, YL and HZhao: writing-review and editing. All authors contributed to the article and approved the submitted version.

## Funding

This work was supported by Natural Science Foundation of Shaanxi Province (2021SF-075), Science and Technology Plan Project of Yulin City (YF-2020-191) and Shenmu Municipal Government Scientific Research Project (2019) No. 5.

## Conflict of Interest

The authors declare that the research was conducted in the absence of any commercial or financial relationships that could be construed as a potential conflict of interest.

## Publisher's Note

All claims expressed in this article are solely those of the authors and do not necessarily represent those of their affiliated organizations, or those of the publisher, the editors and the reviewers. Any product that may be evaluated in this article, or claim that may be made by its manufacturer, is not guaranteed or endorsed by the publisher.
